# Delayed intrinsicoid deflection: Electrocardiographic harbinger of heart disease

**DOI:** 10.1111/anec.12940

**Published:** 2022-02-17

**Authors:** Achilles V. Aiken, Joshua I. Goldhaber, Sumeet S. Chugh

**Affiliations:** ^1^ Cedars‐Sinai Smidt Heart Institute Los Angeles California USA

**Keywords:** delayed intrinsicoid deflection, heart failure, left ventricular hypertrophy, R wave peak time, sudden cardiac death

## Abstract

Delayed intrinsicoid deflection (DID) is an emerging electrocardiogram (ECG) marker of major clinical significance that is increasingly getting attention. Intrinsicoid deflection measures ventricular depolarization in the initial portion of the QRS complex, and DID is defined as an R wave peak time of ≥50 ms in leads V_5_ and V_6_. Prior studies have identified an independent association between DID and cardiovascular conditions such as left ventricular hypertrophy, heart failure, and sudden cardiac death. The exact mechanism that results in DID remains unknown. Animal models indicate that DID may result from abnormal calcium and potassium conductance as well as extracellular matrix remodeling. DID remains an ECG marker of interest given its potential predictive value of underlying cardiovascular pathology and adverse events. This review provides an update on the proposed mechanisms and associations, as well as the clinical and research implications of DID.

## 1 INTRODUCTION

Heart disease is the leading cause of death in the United States and accounts for nearly one out of every four deaths (Virani et al., 2020). Nationwide, an estimated 655,000 people die from heart disease each year (Virani et al., 2020). Sudden cardiac death (SCD) accounts for 300,000 to 330,000 deaths annually (Stecker et al., 2014), and an estimated 379,800 patients who died in 2018 carried a diagnosis of heart failure (Virani et al., 2020). Despite a plethora of diagnostic tools and therapeutic options developed in recent decades, cardiovascular disease continues to account for more deaths in the United States than any other cause. The field continues to require enhancement of screening and preventative modalities with the goal of intervening earlier using broadly available risk assessment methodology.

The electrocardiogram (ECG) offers a readily accessible and cost‐effective method for cardiovascular disease screening. Several ECG markers have been associated with underlying heart disease. For example, prolonged QRS, QTc, and JTc intervals have individually been associated with increased risk of SCD (Algra et al., 1991; Aro et al., 2011; Chugh et al., 2009; Straus et al., 2006; Teodorescu et al., 2011) with more recent development of ECG risk scores to enhance clinical risk prediction (Aro et al., 2017). QRS duration ≥150 ms and the presence of a left bundle branch block (LBBB) are used to identify patients with heart failure and reduced ejection fraction (HFrEF) who may benefit from cardiac resynchronization therapy (CRT) (Tracy et al., 2013). Based on emerging data regarding independent associations with left ventricular hypertrophy (LVH), heart failure, and SCD (Darouian et al., 2016; O'Neal et al., 2016; Romhilt & Estes, 1968), there is an active and growing interest in delayed intrinsicoid deflection (DID) as an ECG marker of specific heart disease conditions. Utilizing DID as a screening tool may provide an opportunity to identify patients with undiagnosed heart disease prior to adverse clinical outcomes, and thus allow clinicians the ability to maximize the benefits of the available cardiac therapies. Given its association with cardiovascular pathology and adverse cardiovascular events, DID may provide a window to identify patients in advance of these cardiovascular sequelae to reduce the mortality burden associated with heart disease.

## 2 DEFINITION AND MEASUREMENT

The term intrinsicoid deflection (ID) is defined as the R wave time to peak and was first reported by MacLeod, Wilson, and Barker in 1930 (Macleod & Barker, 1930). ID represents the initial phase of ventricular depolarization and is the measurement of the time from the onset of the QRS complex to the peak of the R wave just prior to the first downward deflection. If there are multiple R wave peaks, such as in a right bundle branch block (RBBB) in lead V_1_, the ID is measured from the beginning of the QRS complex to the last R wave peak just prior to steepest downward deflection. The normal R wave peak time of the left ventricle is <50 ms (Perez‐Riera et al., 2016). DID is typically defined as an R wave peak time ≥50 ms in leads V_5_ and V_6_ (Sokolow & Lyon, 1949) and represents a delay in the initial phase of left ventricular (LV) depolarization. Since Leads V_5_ and V_6_ are the standard for measurement of ID time, incorrect lead placement could potentially affect the accuracy of this measurement.

## 3 MECHANISMS AND PATHOPHYSIOLOGY

**FIGURE 1:**
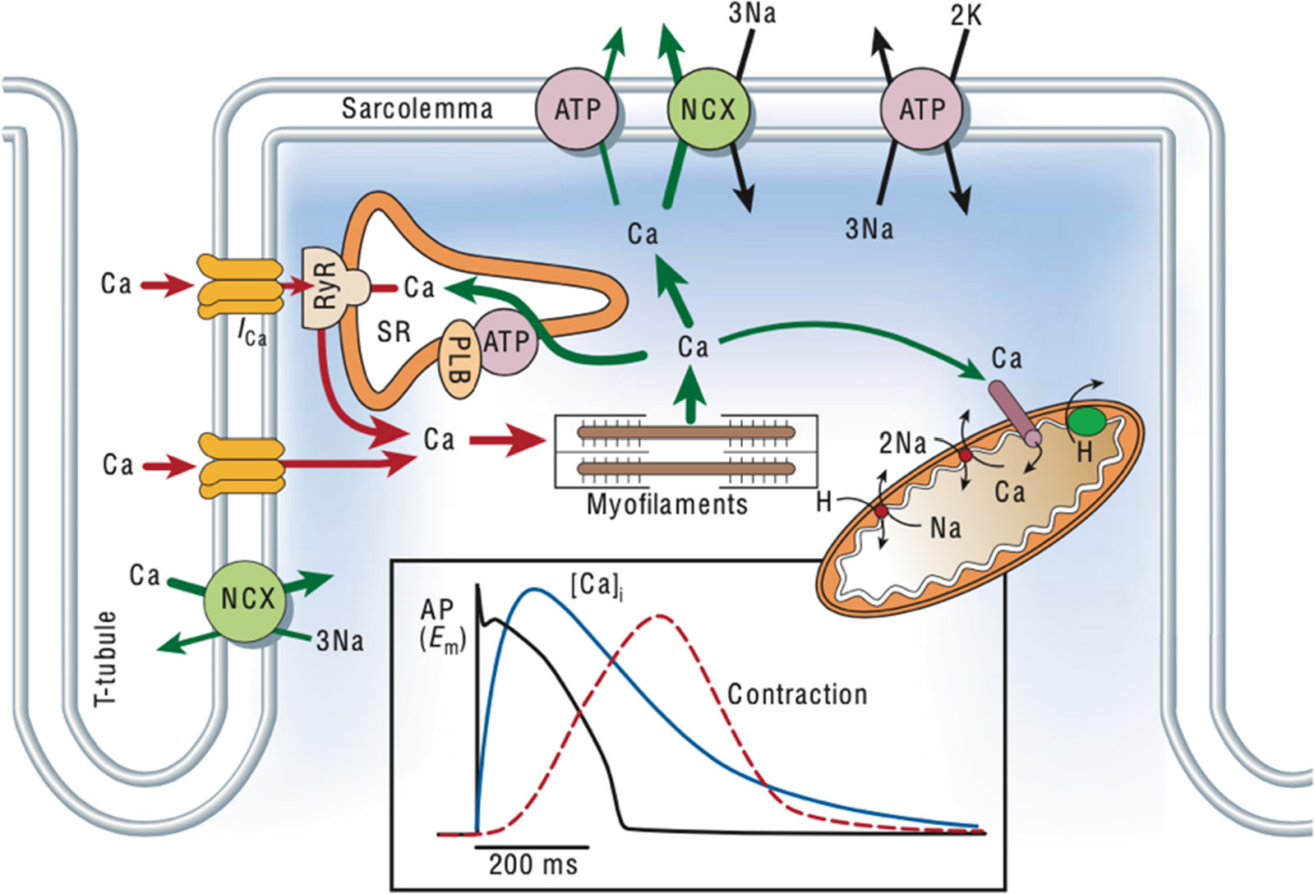
Calcium current during the plateau phase of the action potential. Calcium influx via L‐type calcium channels (I_Ca_) and sodium influx via the sodium‐calcium exchanger (NCX) results continued myocyte depolarization during the plateau phase of the action potential. (Reprinted with permission from Bers, 2002)

Multiple mechanisms have been proposed for the delay in ventricular depolarization observed in DID. One proposed mechanism is attributed to abnormalities in myocardial action potential related to calcium and potassium ion channel function. The action potential duration of the ventricular cardiac myocyte is largely determined by the plateau phase and repolarization phase. During the plateau phase of the action potential, calcium ion influx into the cardiac myocyte via the L‐type calcium channel and sodium ion entry via the Na/Ca exchanger result in continued depolarization of the myocyte (Figure 1) (Bers, 2002), which is balanced by the repolarizing effects of potassium ion efflux from the cardiac myocyte via potassium ion channels. During the repolarization phase, calcium channels are mostly inactive and potassium ion efflux persists resulting in repolarization (Spragg et al., 2018).

**FIGURE 2:**
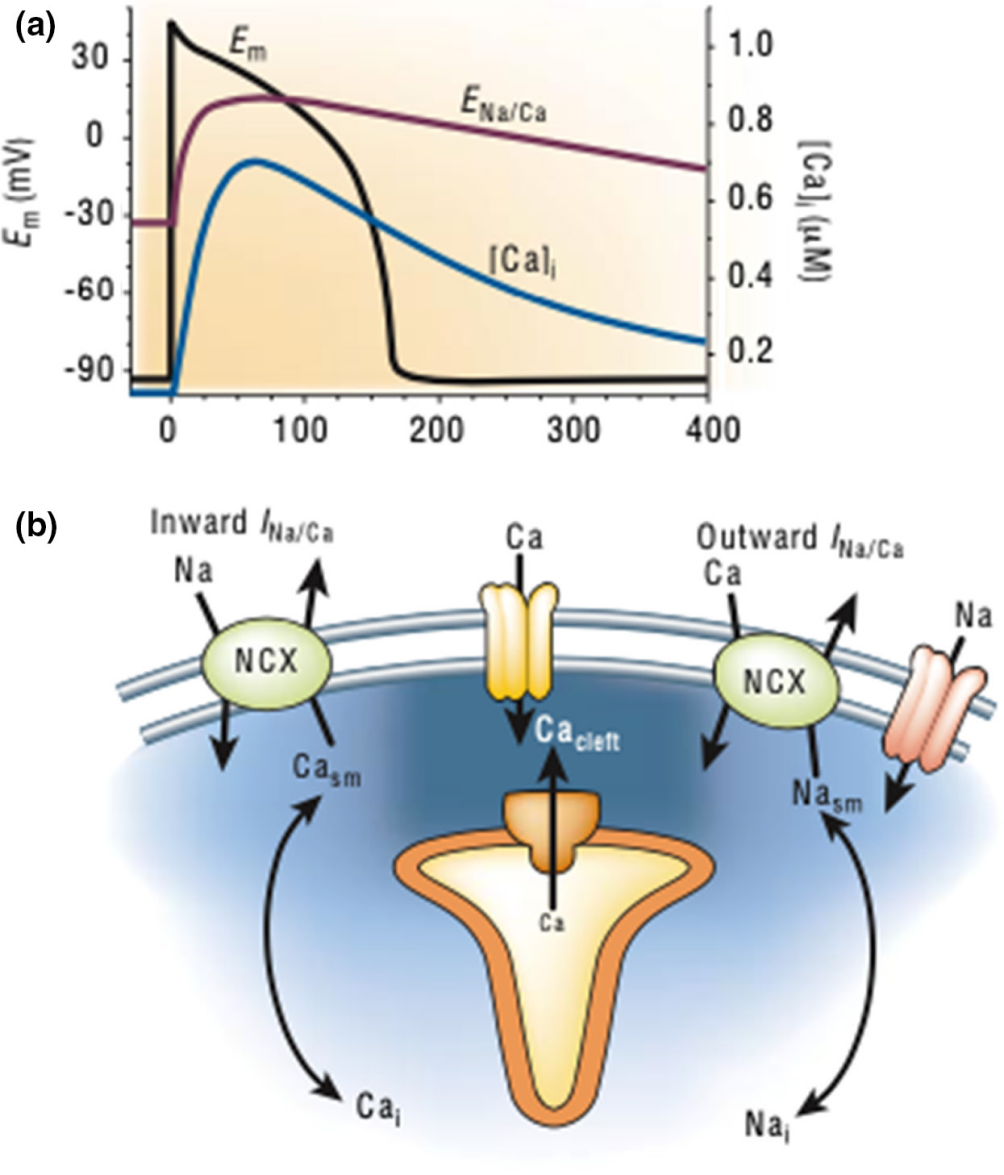
Sodium‐calcium exchanger during an action potential. (a) Calcium current [Ca]i increases during the plateau phase of the action potential and results in increased sodium‐calcium exchanger activation (E_Na/Ca_). (b) Inward current (I_Na/Ca_) via the sodium‐calcium exchanger (NCX) results from sodium influx and calcium efflux. Outward current (I_Na/Ca_) results from sodium efflux and calcium influx. (Adapted with permission from Bers, 2002).

In female guinea pigs undergoing infrarenal aortic coarctation resulting in mild LVH, action potential durations were prolonged when compared to the non‐hypertrophied female guinea pig myocytes. Hypertrophied guinea pig myocytes displayed increased calcium current via increased L‐type calcium current density and prolonged sodium‐calcium exchanger current activation. Prolonged sodium‐calcium exchanger activation during the plateau phase of the action potential results in increased sodium influx and calcium efflux, which prolongs the action potential duration (Figure 2) (Bers, 2002). In this animal model of a mild pressure‐overload system, there was no change in potassium current (Ryder et al., 1993). The increase in calcium current found in guinea pigs with LVH may reveal a component of the electrophysiologic changes that contribute to action potential prolongation via prolonged plateau and repolarization phases resulting in DID.

In guinea pigs with severe LVH, an increase in calcium current was again identified; however, a reduction in potassium current via I_k1_ and I_k_ channels was also observed (Ryder et al., 1991). In rabbits with LVH, reduction in potassium current via decreased I_k1_ and I_to_ potassium channel currents was also found in hypertrophied myocytes along with action potential prolongation when compared to non‐hypertrophied rabbit hearts (McIntosh et al., 1998). In cats with LVH following aortic banding, action potential durations were prolonged, but with increased variability. There was greater dispersion of refractoriness, the difference between the shortest and longest refractory periods, among contiguous hypertrophied cardiac myocytes when compared to cats with non‐hypertrophied cardiac myocytes. The hypertrophied left ventricles of cats also displayed lower excitability thresholds for induction of ventricular fibrillation (VF) when compared to non‐hypertrophied cat left ventricles (Kowey et al., 1991). The increased calcium current and reduced potassium current observed in animal models of LVH may produce prolonged plateau and repolarization phases resulting in action potential prolongation as well as variability in both action potential duration and refractory period. These dispersions of action potential duration and refractory period may produce the substrate that promotes re‐entry circuits and arrhythmogenesis in LVH (Wolk, 2000). These findings may also play a role in the mechanism by which DID is observed clinically in patients with LVH and predisposes patients with LVH to ventricular arrhythmias and SCD.

**FIGURE 3:**
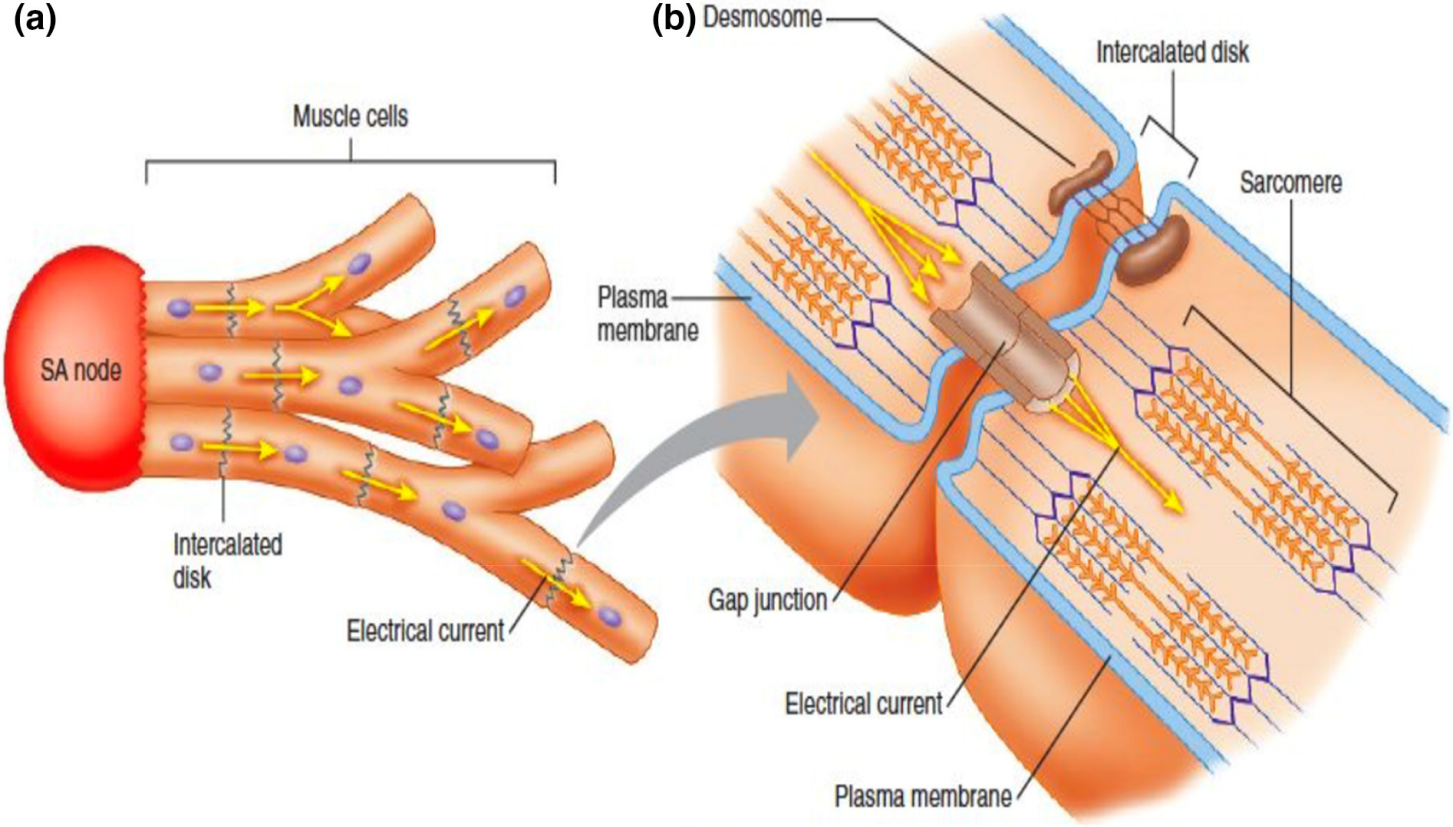
Myocardial gap junctions. (a) An action potential generated in the sinoatrial (SA) node and an electrical current conducted to adjacent myocytes via gap junctions within intercalated disks. (b) A gap junction transmitting an electrical current between adjacent myocytes. (Adapted with permission from Stanfield, 2017)

Another proposed mechanism that may contribute to the development of DID identified in a prior study is the reduction in myocardial cell gap junctions in ischemic and hypertrophied human heart tissue (Peters et al., 1993). Gap junctions are important organelles required for intercellular conductance within the myocardium (Figure 3) (Stanfield, 2017). In this study, surgical myocardial tissue samples from chronically ischemic or hypertrophied ventricular myocardial tissue were sampled and compared to normal ventricular myocardium. The chronically ischemic or hypertrophied ventricular myocardial tissue samples displayed a preserved number of intercalated discs when compared to normal ventricular myocardial tissue. However, both the ischemic and hypertrophied myocardial tissue displayed a 40% reduction in gap junction surface area per unit volume when compared to normal myocardial tissue (Peters et al., 1993). These findings indicate that myocardial remodeling in both the ischemic and hypertrophied heart results in decreased gap junction density between myocytes, which may contribute to the action potential duration prolongation and increased dispersion observed in hypertrophied animal left ventricles. These myocardial abnormalities may contribute to the the arrhythmias and cardiac conduction abnormalities associated with LVH and ischemic heart disease. DID may also manifest electrocardiographically via delayed electrochemical communication between contiguous myocytes as a result of reduced gap junction availability.

**FIGURE 4:**
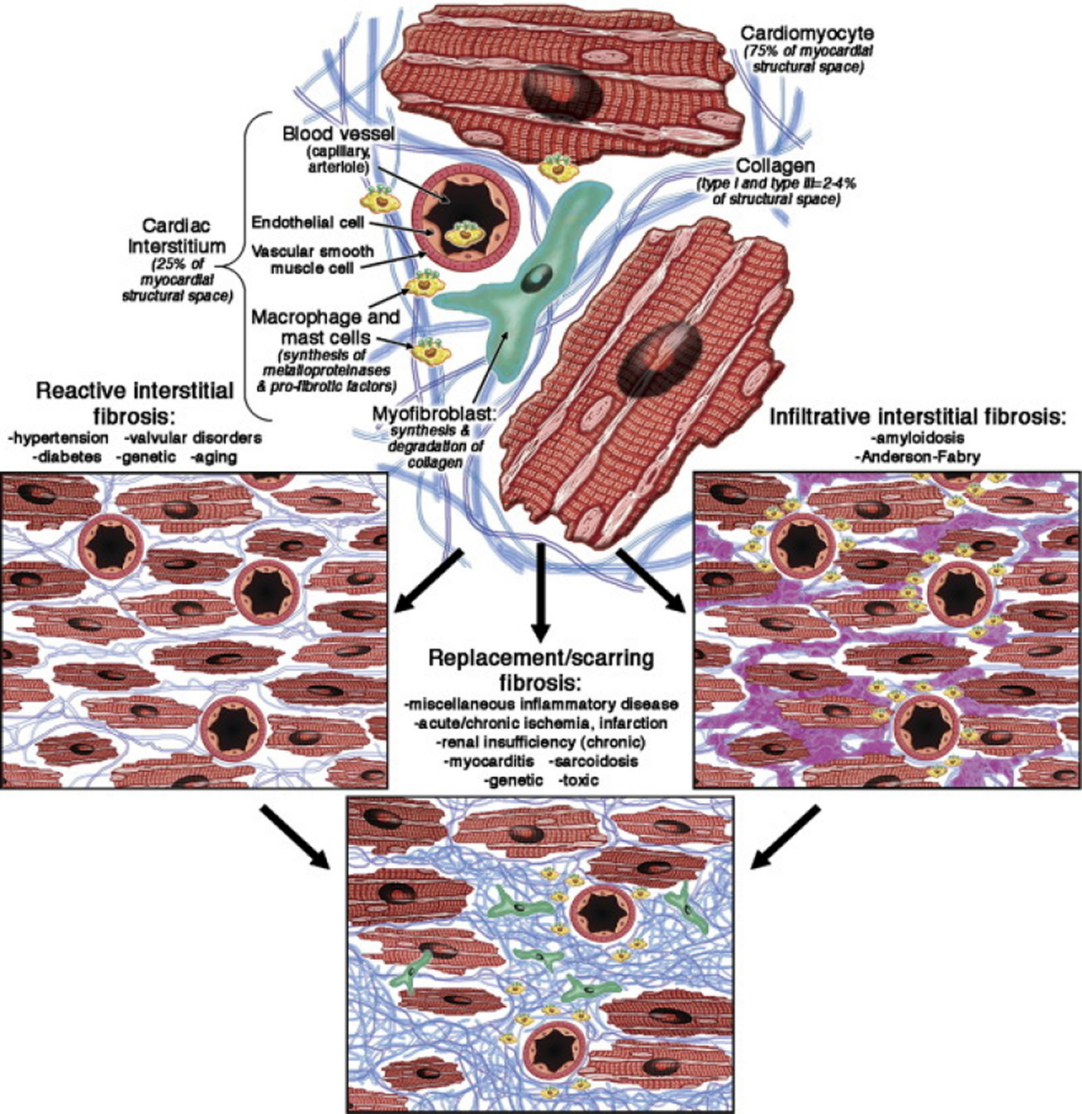
Extracellular matrix remodeling. Myocardial injury leads to abnormal extracellular matrix remodeling in the form of myocyte hypertrophy, interstitial fibrosis, and collagen deposition. (Adapted with permission from Mewton et al., 2011)

The extracellular matrix of the myocardium also appears to play a role in the delayed ventricular depolarization observed in DID (Figure 4) (Mewton et al., 2011). Hypertrophied hearts of primates with perinephritis and systemic hypertension were found to have increased quantity of collagen deposition between myofibers (Weber et al., 1987). A number of studies have also observed that interstitial fibrosis follows collagen deposition in systemic hypertension and myocardial hypertrophy among both primate (Abrahams et al., 1987; Pick et al., 1989; Weber et al., 1988) and rat (Carroll et al., 1989; Doering et al., 1988; Jalil et al., 1988, 1989; Jalil, Doering, et al., 1989; Silver et al., 1990; Weber et al., 1990) models. Myocyte size may also contribute to the electrophysiological alterations seen in LVH especially since conduction velocity was increased yet QRS duration was prolonged in a rabbit model of LVH and heart failure, when compared to normal rabbit hearts (Wiegerinck et al., 2006). Therefore the pathophysiology of DID appears to be multifactorial. In addition to action potential prolongation and increased dispersion via altered calcium and potassium ion conductance; reduced gap junction density, increased interstitial fibrosis and increased cardiac myocyte size may also contribute to DID.

## 4 LEFT VENTRICULAR HYPERTROPHY

Delayed intrinsicoid deflection appears to represent an ECG marker of an underlying conduction defect that has been independently associated with multiple cardiovascular disease processes. DID ≥50 ms in leads V_5_ and V_6_ has been associated with LVH and is used in the Romhilt‐Estes (R‐E) ECG criteria for LVH (Noth et al., 1947; Romhilt et al., 1969; Sokolow & Lyon, 1949). The Romhilt‐Estes (R‐E) ECG criteria for LVH has also been associated with an increased all‐cause mortality (Estes et al., 2015). In previous studies, DID was attributed to increased left ventricular (LV) mass and displayed a positive correlation with LV mass (Baxley et al., 1968; Grubschmidt & Sokolow, 1957). Despite the association of DID and the R‐E ECG criteria with LVH, the association between DID and echocardiographic LVH has been inconsistent (Darouian et al., 2016). In fact, the sensitivity of the R‐E ECG criteria for predicting increased LV mass has been estimated at only 60% (Romhilt & Estes, 1968). Previous studies have also reported that the R‐E ECG score predicts sudden cardiac arrest (SCA) independent of LV mass and ejection fraction (Darouian et al., 2017). As such, there appears to be an overlap between the association of DID with ECG LVH and anatomic LVH; however, DID appears to confer an arrhythmogenic risk independent of anatomic LVH as well.

## 5 HEART FAILURE

Abnormalities in ventricular depolarization as measured by ECG markers have been associated with development of heart failure. QRS prolongation as well as the presence of left bundle branch block (LBBB), as opposed to right bundle branch block (RBBB), have been associated with the incidence of heart failure (Ilkhanoff et al., 2012; Zhang et al., 2015). A previous study also identified an association between increasing ID time and risk for heart failure events. In this study, for each 10 ms increase in ID time, there was a 1.42 greater risk of heart failure events, and that risk increased significantly when the ID time was >45 ms (O'Neal et al., 2016). Given the stronger association between LV conduction defects and heart failure, DID may be a better predictor of delayed LV activation, and therefore heart failure, compared to QRS duration. DID appears to not only predict the risk of heart failure, but also the preferential risk of HFrEF over HFpEF. In another study, DID of >50 ms in leads V_5_ and V_6_ was a strong predictor of heart failure events (HR 2.81). However, only the risk of HFrEF (HR 4.90) events was strongly associated with DID, with no signficiant association identified between DID and the risk of HFpEF (HR 0.94). HFpEF events were instead associated with abnormal P‐wave axis, QRS‐T axis, and higher resting heart rate (O'Neal et al., 2017). These findings imply that distinct ECG markers arising from likely distinct pathophysiological mechanisms, appear to predict HFrEF and HFpEF.

In patients with HFrEF, QRS prolongation and the presence of a LBBB are utilized to guide the implementation of CRT (Tracy et al., 2013). However, up to one‐third of HFrEF patients do not respond to CRT despite its potential reverse remodeling (RR) and mortality benefits (Auricchio & Prinzen, 2011; Prinzen et al., 2013). QRS duration is a measure of both right and left ventricular conduction, which may limit its utility in predicting CRT response (Sipahi et al., 2011). DID is another ECG marker that has been associated with response to CRT. Prolonged time to ID in lateral leads, I and aVL, was found to be a better predictor of volumetric RR response to CRT than pre‐implantation QRS duration or post‐implantation QRS reduction (Del‐Carpio Munoz et al., 2013). DID may identify patients who are at risk for developing heart failure, and also improve selection HFrEF patients who are more likely to benefit from heart failure therapies such as CRT.

## 6 SUDDEN CARDIAC DEATH

ECG markers, including prolonged QRS, QTc, JTc, and elevated resting heart rate have been associated with SCD (Algra et al., 1991; Aro et al., 2011; Chugh et al., 2009; Straus et al., 2006; Teodorescu et al., 2011). LVH, by both electrical and anatomic measurements, has also been associated with an increased risk of SCD (Romhilt & Estes, 1968). DID of ≥50 ms in leads V_5_ and V_6_ was found to be an independent risk factor for SCA with an odds ratio of 1.82 when controlled for diabetes, chronic renal insufficiency, severe LV dysfunction (LVEF ≤35%), echocardiographic LVH, QRS duration, JTc prolongation, and heart rate (Darouian et al., 2016). LVEF, as an isolated risk factor, has been associated with an arrhythmic mortality risk of less than 5% over a 2‐year span (Buxton et al., 2007). Despite the association of SCA with multiple other markers such as DID, LVEF continues to be the main parameter by which implantable cardioverter‐defibrillator (ICD) therapy is offered to heart failure patients for primary prevention of SCA (Al‐Khatib et al., 2018). DID appears to indicate an underlying myocardial abnormality that is independent of currently used diagnostic measures, and warrants further evaluation as a clinical predictor of SCA.

## 7 CLINICAL AND RESEARCH IMPLICATIONS

**FIGURE 5:**
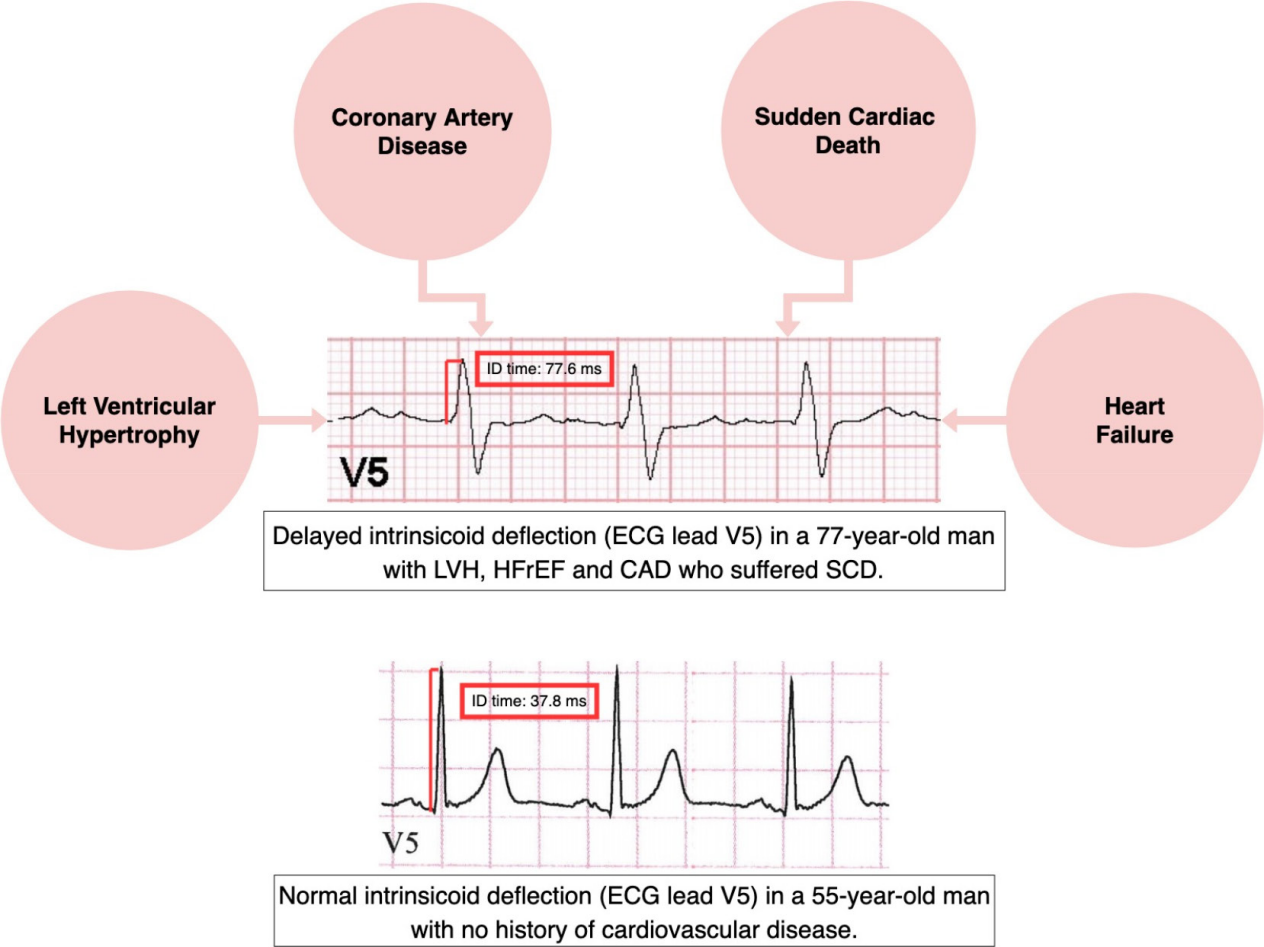
Clinical examples of delayed and normal intrinsicoid deflection linked to specific clinical conditions. Delayed intrinsicoid deflection (DID) is an electrocardiographic (ECG) manifestation of underlying cardiovascular disease including left ventricular hypertrophy (LVH), coronary artery disease (CAD), heart failure (in this case HFrEF, heart failure with reduced ejection fraction), and sudden cardiac death (SCD)

Given the association of DID with underlying heart disease, identifying DID in patients at risk for these cardiovascular pathologies may offer clinicians a window to prevent the cardiac complications associated with DID or influence the clinical trajectory of patients whose pathology has already manifested (Figure 5). In patients with coronary artery disease (CAD) and angina without typical ECG or serological findings consistent with ongoing myocardial ischemia, DID may potentially indicate increased risk of future clinical events warranting more urgent ischemic evaluation, and should be evaluated further. In patients with essential hypertension, the development of DID could be an indicator of LVH and the need for more aggressive lifestyle modifications and antihypertensive medical management as well as monitoring for clinical symptoms of impending heart failure. In LVH and diastolic heart failure, Q waves in the left lateral precordial leads V_5_ and V_6_ result in increased ID time (Perez‐Riera et al., 2016). DID monitoring may also provide the opportunity to identify currently asymptomatic patients who are at risk for developing clinical HFrEF. DID also offers the potential to improve prediction of which patients would benefit from CRT.

While additional prospective studies are warranted, there is likely enough evidence in the existing literature to prompt a cardiac evaluation in patients who are found to have DID on the 12‐lead ECG. Given that for each 10 ms increase in ID time there is a 1.42 greater risk of heart failure events (O'Neal et al., 2016), we suggest consideration of clinical evaluation in patients with DID of 50 ms or greater. Even if the finding is incidental, it is reasonable to assess cardiovascular risk factors, but need for a coronary disease evaluation and assessment of cardiac structure and function with an echocardiogram is warranted. Depending on the nature and severity of symptoms as well as the clinical presentation, this workup could be escalated and/or broadened due to an increased pre‐test probability of coronary artery disease and heart failure. Specifically for sudden cardiac death risk stratification, this marker has significant potential, but larger prospective studies are needed prior to clinical adoption.

Since intrinsicoid deflection is a “component” of the QRS interval, there is likely a relationship between these two measurements although there is limited data in the literature specifically evaluating this relationship. However, as best as we can tell, the two markers may also have distinct associations depending on the disease condition being evaluated. In the context of sudden cardiac death, DID appears to be a better predictor than QRS duration alone. DID has been independently associated with SCA when controlling for heart rate, QRS duration, severe LV dysfunction, echocardiographic LVH, diabetes mellitus, and chronic renal insufficiency. QRS duration was not independently associated with SCA in the same multivariate analysis (Darouian et al., 2016). In heart failure, there appears to be more of an overlap with both DID (HR 2.81) and prolonged QRS (HR 1.70) appearing to predict heart failure events. DID also confers a stronger association with HFrEF (HR 4.90) as opposed to HFpEF (HR 0.94) (O'Neal et al., 2017). In the context of selecting candidates for CRT, the vast majority of studies suggest that QRS duration is a clinically effective marker. However, prolonged time to ID in lateral leads, I and aVL, was found to be a better predictor of volumetric RR response to CRT than pre‐implantation QRS duration (Del‐Carpio Munoz et al., 2013). Overall, this is likely a complex relationship between DID and QRS duration, and future studies should make the effort to evaluate both markers.

The nationwide mortality burden attributed to SCD remains high despite efforts at primary prevention with ICD therapy. This disparity may be due to the use of severely reduced LVEF ≤35% as the main parameter by which ICD therapy is offered for primary prevention in heart failure, since there is estimated to only be a 2–5% annual risk of SCD among patients with an LVEF ≤35% and only one‐third of SCD cases having an LVEF ≤35% (Bardy et al., 2005; Moss et al., 2002; Stecker et al., 2006). Extending beyond LVEF as the sole marker by which ICD therapy is offered for primary prevention in heart failure, and utilizing other markers, such as DID, could result in improved candidate selection for the primary prevention ICD.

More research is needed to further elucidate the mechanisms by which DID reflects evolving myocardial conduction disease that places patients at risk for adverse cardiovascular events. Large, detailed studies that evaluate the association of DID with LVH, heart failure, and SCA should improve the predictive value of DID for these manifestations of heart disease. This could lead to development of enhanced screening strategies that facilitate preemptive interventions for overall reduction of cardiovascular morbidity and mortality.

## 8 PERSPECTIVES

- DID is an emerging ECG marker associated with multiple adverse cardiovascular events including SCD.
- DID manifests phenotypically in LVH, CAD, and HFrEF patients.
- DID may reflect abnormal calcium and potassium conductance and extracellular matrix remodeling.
- Further research is needed to incorporate DID in risk stratification strategies that enhance prevention of adverse cardiovascular events.

## AUTHOR CONTRIBUTIONS

AVA initial draft and completion of manuscript; JIG review and revisions to manuscript; SSC conception, review and revisions to manuscript.

## ETHICAL APPROVAL

Does not meet criteria for review by institution review board. Literature search data available upon request.

## CONFLICT OF INTEREST

The authors declare no conflicts of interest.
